# Renal Cell Carcinoma in von Hippel–Lindau Disease—From Tumor Genetics to Novel Therapeutic Strategies

**DOI:** 10.3389/fped.2018.00016

**Published:** 2018-02-09

**Authors:** Emily Kim, Stefan Zschiedrich

**Affiliations:** ^1^Department of Radiation Oncology, Faculty of Medicine, Albert Ludwigs University of Freiburg, Freiburg, Germany; ^2^German Cancer Consortium (DKTK), Partner Site Freiburg, Freiburg, Germany; ^3^Renal Division, Department of Medicine IV, Faculty of Medicine, Albert Ludwigs University of Freiburg, Freiburg, Germany

**Keywords:** von Hippel–Lindau disease, renal cell carcinoma, cancer genetics, predictive biomarkers, preclinical models, new therapeutic targets

## Abstract

von Hippel–Lindau (VHL) disease is an autosomal dominant syndrome caused by mutations in the VHL tumor-suppressor gene, leading to the dysregulation of many hypoxia-induced genes. Affected individuals are at increased risk of developing recurrent and bilateral kidney cysts and dysplastic lesions which may progress to clear cell renal cell carcinoma (ccRCC). Following the eponymous *VHL* gene inactivation, ccRCCs evolve through additional genetic alterations, resulting in both intratumor and intertumor heterogeneity. Genomic studies have identified frequent mutations in genes involved in epigenetic regulation and phosphoinositide 3-kinase–AKT–mechanistic target of rapamycin (mTOR) pathway activation. Currently, local therapeutic options include nephron-sparing surgery and alternative ablative procedures. For advanced metastatic disease, systemic treatment, including inhibition of vascular endothelial growth factor pathways and mTOR pathways, as well as immunotherapy are available. Multimodal therapy, targeting multiple signaling pathways and/or enhancing the immune response, is currently being investigated. A deeper understanding of the fundamental biology of ccRCC development and progression, as well as the development of novel and targeted therapies will be accelerated by new preclinical models, which will greatly inform the search for clinical biomarkers for diagnosis, prognosis, and response to treatment.

## Introduction

von Hippel–Lindau (VHL) disease is an autosomal dominant, multiorgan visceral cysts and tumor syndrome. The disease name derives from the German ophthalmologist Eugen von Hippel who studied two cases of striking retinal angiomas and the Swedish pathologist Arvid Lindau who detected a connection between cerebellar hemangioblastomas, retinal angiomas, and other visceral tumors (1, 2). The first report of VHL dates from 1894, when Collins described vascular intraocular tumors in two siblings (3). VHL manifestations can be found in retinal hemangioblastoma, cerebellar and spinal hemangioblastoma, renal cysts and clear cell renal cell carcinoma (ccRCC), liver hemangioma, pancreatic cysts, pancreatic microcystic serous adenoma and pancreatic neuroendocrine tumors, pheochromocytoma (PCC), epididymal and broad ligament cystadenoma, and endolymphatic sac tumors. In 1964, Melmon and Rosen suggested clinical diagnostic criteria that are still valid today. VHL disease has been classified depending on the presence of PCC in VHL type 1 (without PCC) and type 2 (with PCC). VHL type 2 is further subclassified in type 2A (with PCC but without ccRCC), type 2B (with PCC and ccRCC), and type 2C (PCC only) (4–6). Practically, however, practitioners do not rely on this classification because families can move between clinical subtypes.

von Hippel–Lindau disease is caused by mutations in the *VHL* tumor-suppressor gene, located on chromosome 3p25-26. *VHL* was mapped to chromosome 3 in 1988 and cloned in 1993 (7, 8). The incidence of VHL disease is approximately 1:36,000 (9). Although VHL disease typically presents in early adulthood, manifestation of retinal angiomas, PCCs, and ccRCCs has been reported earlier; therefore, guidelines recommend starting surveillance programs for eye examination at 2–5 years and abdominal imaging at 6–10 years (10–12).

Most patients develop ccRCCs that arise from microscopic precursor lesions present in both kidneys. The number of nonmalignant cysts lined with clear cells in an average VHL kidney was estimated to be 1,100, and the number of clear cell renal neoplasms (solid and cystic) to be 600 (13). Optimally, early RCC can be detected, observed, and surgically removed before progression to metastatic disease (14). However, repeated surgery for multifocal bilateral disease is followed by increased risk of end-stage renal disease (ESRD) requiring renal transplantation or dialysis.

## The Role of VHL

Whether hereditary or sporadic, ccRCC is characterized by mutations in the VHL tumor-suppressor gene on chromosome 3 (3p25-26) and a subsequent loss of heterozygosity. VHL, Elongin B (encoded by *TCEB1*), and Elongin C form a stable complex that targets hypoxia-inducible factor α (HIFα) subunits for proteolytic degradation under normoxic conditions (15). In the presence of hypoxia or in the absence of functional VHL tumor-suppressor protein, HIFα subunits HIF1α and HIF2α are stabilized, binding together with ARNT (HIF1β) to hypoxia-response elements to activate genes involved in angiogenesis, cell cycle, cell proliferation, glucose, and lipid metabolism, among others. Mutations of *TCEB1* that abrogate binding of Elongin B to VHL can also increase HIFα expression in ccRCCs with intact *VHL* (16). Mutations in *VHL* and *TCEB1* were mutually exclusive, supporting a permissive role for VHL complex degradation and HIFα stabilization in tumorigenesis.

## Additional Gene Mutations

Approximately 15 years after *VHL* was identified as the genetic basis for VHL, further driver mutations for ccRCC were identified, summarized in Table 1. Exome sequencing of tumors identified additional inactivating mutations on chromosome 3 in tumor suppressors polybromo-1 (*PBRM1*), SET domain-containing 2 (*SETD2*), and BRCA1-associated protein-1 (*BAP1*), which are chromatin and histone regulators located at 3p21 (17–19). *PBRM1* and *BAP1* mutations are mutually exclusive, with *BAP1* mutations correlating with higher grade disease (19, 20). Large-scale genomic sequencing showed that these three tumor-suppressor genes, located near *VHL* at 3p21, are the most frequently mutated genes in ccRCC after *VHL* (16, 21). Other significantly mutated genes included histone-modifiers *KDM5C* and *KDM6A*, previously implicated in ccRCC (17–19, 22), genes in the phosphoinositide 3-kinase (PI3K)/AKT pathway, such as mechanistic target of rapamycin (*mTOR*), *PIK3CA*, and *PTEN*, as well as *TCEB1* (Elongin C) (16, 21). The tumor-suppressor gene *TP53* is infrequently mutated, playing a lesser role than in many other solid tumors.

**Table 1:** Frequently mutated genes in clear cell renal cell carcinoma (16, 21, 23).

Gene	Chromosome	Protein	Function	Mutation (%)
VHL	3p25.3	von Hippel–Lindau (VHL) disease	VHL, Elongin B and C complex	49–82
PBRM1	3p21.1	Polybromo 1	SWI–SNF complex chromatin remodeling	29–41
SETD2	3p21.31	SET domain-containing 2	Histone H3K36 methyltransferase	8–12
BAP1	3p21.1	BRCA1-associated protein 1	Histone deubiquitinase	6–10
KDM5C	Xp11.22	Lysine demethylase 5C (JARID1C)	H3K4 demethylase	4–8
mTOR	1p36.22	Mechanistic target of rapamycin kinase	Phosphoinositide 3-kinase (PI3K)–AKT–mechanistic target of rapamycin (mTOR) pathway	5–6
PTEN	10q23.31	Phosphatase and tensin homolog	PI3K–AKT–mTOR pathway	4
PIK3CA	3q26.32	PI3K catalytic subunit α	PI3K–AKT–mTOR pathway	3–5
TP53	17p13.1	Tumor protein P53	Cell cycle	2–3
TCEB1 (ELOB)	16p13.3	Elongin B	VHL, Elongin B and C complex	1–3

*Loss of heterozygosity at 3p was reported in over 90% of cases, and mutations in components of the PI3K–AKT–mTOR pathway in 28–76%*.

## Tumor Heterogeneity

Clear cell renal cell carcinoma has a moderate somatic mutation frequency compared with other solid tumors and these mutations progress in a branched evolutionary manner (24, 25). The evolutionary history of sporadic ccRCC in 10 individuals was investigated by exome sequencing of multiregion samples from primary ccRCCs and metastases (26). Intratumor heterogeneity was present in all tumors, indicating that a single biopsy underestimates the genomic complexity of a tumor. Tumor phylogeny, similar to an evolutionary tree, showed that inactivation of *VHL* and loss of chromosome 3p were ubiquitous early truncal events. *PBRM1* inactivation was a frequent mutation, occurring early as a truncal mutation in three tumors and as a later event in three others. Distinct subclones, spatially separated within a single tumor, contained mutations that appeared in a branched rather than linear manner. Subclonal driver mutations were similar to those identified by earlier studies, such as chromatin modifiers, regulators of mTORC1 pathway, and tumor-suppressor *TP53*. Parallel evolution was observed in certain genes, in which different evolutionary paths or branches in a tumor resulted in inactivation of the same gene by separate mechanisms. Recurrent mutations in *PBRM1, SETD2, BAP1*, and *KDM5C* suggest an evolutionary selection for epigenetic dysregulation in tumorigenesis. Branched subclonal mutations were highly variable and contained more C>T transitions than truncal mutations, potentially useful as prognostic and predictive biomarkers.

Genomic analysis of ccRCC has established the fundamental role for VHL inactivation and HIF dysregulation, the importance of chromatin regulation and histone modification, and the involvement of the mTORC1 pathway. The central role of chromosome remodeling in the development and the progression of ccRCC implicates epigenetic dysregulation as a permissive factor in tumorigenesis and a novel target for therapeutic agents and candidate biomarkers. However, the molecular mechanisms whereby epigenetic alterations result in transcriptional dysregulation is currently unclear. Mutations and copy number alterations were detected in *mTOR* (mammalian target of rapamycin), *PIK3CA* (PI3K catalytic subunit-α), TSC1, and *PTEN* (16, 21). Mutually exclusive gene alterations of the PI3K/Akt/mTOR pathway were detected in approximately 28% of tumors (21). Activation of the PI3K/Akt/mTOR pathway may underlie the efficacy of mTOR inhibitors, such as everolimus and temsirolimus.

In kidneys of individuals with VHL disease, VHL-deficient lesions with constitutive HIF activation were detectable by carbonic anhydrase IX staining, allowing the progression from single cells to ccRCC to be easily observed (27). HIF activation occurs extremely early in the disease. Most lesions are single cells, with very few multicellular dysplastic lesions and cystic lesions, showing that loss of VHL function alone is insufficient for ccRCC formation (27). Renal cysts are classified as benign, atypical, and malignant. HIF-1α is expressed in all VHL-deficient renal cells, whereas HIF-2α is highly expressed in renal tubular cysts and ccRCC (27).

Multiregion whole exome sequencing of four tumors from one individual with VHL disease delineated multifocal tumors of independent clonal origins (28). Each tumor exhibited a loss of chromosome 3p, each with a distinctly different breakpoint. Tumor evolution was more linear than branching compared with sporadic ccRCC, with markedly less intratumor heterogeneity. Convergent mTOR pathway activation was observed in all tumors through distinct gene mutations. The evolutionary history of 40 tumors from 6 individuals with VHL was examined by whole-genome sequencing (29). Tumors showed more genetic homogeneity than sporadically occurring tumors, which are generally removed at a later stage. However, the lack of overlapping sets of single-nucleotide variants as well as copy number variants between tumors indicated that ccRCCs evolved independently. A similar approach with different *VHL* subtypes could elucidate the effect of genetic background on the disease. For example, type 1 VHL disease (without PCC) is associated with truncation or exon deletion of germline VHL, whereas missense mutation is associated with type 2 disease (with PCC) (30). ccRCC occurs in Types 1 and 2B, which poorly downregulate HIF-1α but not 2A and 2C (6, 31).

## Preclinical Models

Tumor xenografts using human ccRCC cell lines or tissue have been extensively used in mice to evaluate potential therapies. Likewise, injecting zebrafish with patient-derived xenografts and human cell lines is a rapid, low-cost preclinical model system of cancer (32, 33). Genetically engineered animal models of biallelic mutation of *VHL* alone in both mouse and zebrafish recapitulate features of early human disease, but not the formation of ccRCC. HIF activation appears to be necessary, but not sufficient for tumor formation in animal models of ccRCC.

Initial animal models were developed by genetically modifying levels of VHL and HIFα. Homozygous deletion of *Vhl* is embryonic lethal at 10.5–12.5 days in mice due to defective placental vasculogenesis; heterozygous mice fail to develop kidney tumors (34). Kidney-specific inactivation of *Vhl* is insufficient for ccRCC development, but results in multiple cysts with constitutive HIF-α expression and metabolic alterations marked by lipid and glycogen accumulation similar to early human disease (35). Transgenic overexpression of HIF-1α or HIF-2α resulted in simple cysts (36, 37). Likewise in zebrafish embryos, homozygous inactivation of *VHL* (*vhl^−/−^*) results in a kidney with enlarged proximal pronephric tubules, disorganized cilia, accumulated lipid and glycogen, cell proliferation, and apoptosis. This phenotype was rescued by a specific HIF2α inhibitor, showing that the zebrafish model system could be used to facilitate rapid screening of candidate drugs (38).

The identification of additional mutations underlying ccRCC has informed the development of genetically engineered mouse models that are more analogous to human disease. After *VHL*, the most frequently mutated genes in ccRCC were chromosome and histone regulators *PBRM1, SETD2*, and *BAP1* (16, 21). Epigenetic genes were targeted in recent studies. Kidney-specific dual inactivation of *Vhl* and *Bap1* or *Pbrm1* using *Pax8-Cre* in mice recapitulated human ccRCC with cytoplasmic accumulation of glycogen and lipids (39). Bap1-deficient cystic tumors were high grade, whereas Pbrm1-deficient solid tumors showed a longer latency. Pbrm1-deficient tumors were converted from low to high grade by disruption of one *Tsc1* allele, resulting in mTORC1 activation. Intriguingly, ccRCC appeared to arise from Bowman capsule cells rather than the proximal tubule based on gene inactivation using more specific *Pax8-Cre* drivers.

Kidney-specific inactivation of *Vhl* and *Pbrm1* using *Ksp-Cre* model the histopathological and molecular features, and gradual onset of human ccRCC (40). *Ksp-Cre* is expressed both in renal tubular cells and the Bowman capsule. Bilateral, multifocal tumors were marked by the clear cytoplasm, high glycogen, and carbonic anhydrase IX staining similar to human ccRCC. A stepwise progression was observed from normal to cystic lesions over 6 months, developing into multifocal ccRCC at ~10 months. Loss of PBRM1 further amplified the activation of HIF1 (hetereodimer HIF-1α and HIF-1β) and STAT3 pathways caused by loss of *Vhl*. Activation of mTORC1 was implicated as the third event leading to ccRCC.

Inactivation of *Vhl, Rb1*, and *Trp53* in mice induced precursor cysts and gradually developing ccRCC tumors (41). Mutations were observed in genes involved in the primary cilium, *Kif3a* and *Kif3b*. Transcriptional analysis showed a gene expression profile similar to that observed in human ccRCC, with upregulation of HIF-1α and HIF-2α, mTOR activity. Mouse tumors showed variable response to anti-angiogenic therapy, and partial response to acriflavine, which interferes with the dimerization of HIF-1α and HIF-2α.

Zebrafish with multiple mutations of VHL disease-related genes are another potential preclinical model for exploring the progression and metastasis of ccRCC with the advantage of live imaging.

## Current Treatment Strategies

### Active Surveillance

Clear cell renal cell carcinomas grow slowly and small tumors <3 cm are at low risk to metastasize in VHL (42, 43). Currently, active surveillance until a threshold size of 3–4 cm is recommended for surgical intervention (43–46), resulting in a recurrence-free survival rate of 76% at 5 years and 20% at 8 years (47).

Regular screening is advised to detect RCC at an early stage and small tumors are followed with serial imaging. To improve the quality of life and survival of these patients, a balance between two goals is paramount: preventing metastases and preserving renal function. The goal is to treat before the tumor metastasizes, but to minimize consequences of the treatment such as compromised renal function. Through better surveillance by regularly scheduled imaging, individuals are living longer; however, a longer lifespan increases the probability of developing multiple RCCs and other sequelae of the disease.

### Treatment of Localized Disease

#### Surgery

Early RCC can be detected, observed, and surgically removed before progression to metastatic disease (14, 48). However, repeated surgery for multicentric bilateral disease is followed by increased risk of ESRD requiring renal replacement therapy. Nephron-sparing surgery at a tumor size of 3–4 cm is the current treatment standard, replacing radical nephrectomy which compromised renal function and resulted in early dialysis. However, repeated partial nephrectomy reduces renal function, eventually causing ESRD. Delaying the interval to kidney surgery without increasing the risk of metastases prolongs sufficient renal function and delays dialysis. Independently of VHL disease, chronic kidney disease is associated with increased risk of death, cardiovascular events, and hospitalization (49). Progression to ESRD not only impacts quality of life but further increases morbidity—the yearly mortality rate of patients receiving long-term dialysis is 15–20% (50).

Locally recurrent disease is not uncommon after both partial nephrectomy and ablative therapy. Therapy options include observation, initial or repeat ablation, initial or repeat partial nephrectomy, radical nephrectomy, or systemic therapy (51). Salvage operation has a high major complication rate approaching 20% (52).

As renal surveillance of VHL patients has shifted surgical treatment from the resection of large tumors to the management of multiple small asymptomatic tumors, nephron-sparing therapeutic options are increasingly used. These minimally invasive procedures include percutaneous radiofrequency ablation (RFA), microwave ablation (MWA), cryoablation, and stereotactic body radiotherapy (SBRT) which are compared with surgery in Table 2.

**Table 2:** Local treatment modalities for clear cell renal cell carcinoma, including indication, frequent complications and limitations of surgery, and ablation techniques.

Treatment	Indication	Frequent complications	Limitations
Nephron-sparing surgery	Gold standard procedure	Bleeding, urinary fistula	Renal insufficiency (especially with repeated interventions)
Radiofrequency ablation	Tumor diameter <4 cm	Perirenal hematoma, postoperative fever, gross hematuria, seldom intraabdominal fistula, bowel injury	Large vessels draw in thermal energy. Large tumors
Microwave ablation	Tumor diameter <4 cm, possible in centrally located tumors	Hematuria, numbness, flank pain, thermal injury, urine fistula, subcapsular renal hemorrhage, seldom urinoma, abscess	Large tumors
Cryoablation	Tumor diameter <4 cm	Bleeding, cardiopulmonary decompensation, myocardial infarct, utero-pelvic junction obstruction	Centrally located tumors
Stereotactic body radiation	Tumor diameter 2–3 cm, not limited by tumor proximity to vessel or collecting system	Nausea, fatigue, skin rash, local pain	Nearby organs of risk (intestine, stomach) require careful planning

#### Radiofrequency Ablation

Initially a treatment for non-surgical candidates, RFA has been used as a first-line procedure used to treat smaller and less numerous stage T1a lesions, and for renal salvage (46). Tumor necrosis is achieved with the heat of 50–100°C of radiofrequency energy transmitted by one or multiple needles in the tumor tissue. Placement of these needles can be achieved both laparoscopically or percutaneous (51).

Short-term local recurrence rates of RFA compare favorably with partial nephrectomy. A study of RFA treated T1 RCC reported a 13% retreatment rate for residual disease, a recurrence-free survival of 94%, and disease-free survival of 88% at 5 years (53). A study of T1a tumors treated with RFA versus partial nephrectomy reported a recurrence-free survival of 91.7 versus 94.6%, and an identical disease-free survival of 89% at 5 years (54). A recent study of 20 RCCs of 1–4 cm diameter in 9 VHL patients treated with RFA showed no recurrence and preserved kidney function with a median follow-up of 102 months (55). Zagoria and colleagues concluded that long-term tumor control could be achieved in lesions treated smaller than 4 cm diameter (56). Another group reported increased risk of residual tumor for lesions >3.5 cm diameter (57). Clearly, there is a decrease of disease-free survival with every centimeter increase of the lesion (56). Although RFA is performed in many centers, conclusions about long-term efficacy are limited by the relatively short follow-up interval and small study sizes.

#### Microwave Ablation

Electromagnetic microwaves, transmitted from one or multiple antennas placed in the tumor can create a thermal field that causes tumor tissue necrosis (58). The antennas can be placed percutaneously, laparoscopically, or less often in an open approach. The potential benefit of Microwave Ablation (MWA) compared with RFA is that intratumoral temperatures with MWA are less affected by the heat sink effect, since MWA is less dependent on the electrical conductivity of the tissue. Larger tumors can be ablated with MWA compared to RFA; therefore, a maximum diameter of 4 cm was recommended in most studies (59–62). Several authors suggest MWA can be performed in tumors close to renal sinus or collecting system—a clear advantage over the other ablation techniques (62, 63).

#### Cryoablation

Cryoablation either laparoscopic or percutaneous, is a minimally invasive procedure that freezes and destroys small tumors (64–67). The cryoprobe is cooled down to −185 to −195°C by a nitrogen-based liquid guided through the tip of the probe. Cryoablation is less precise than RFA as it requires three applicators and a tumor margin of 10 mm. Thus, RFA of a 2-cm mass ablates approximately 10 cm^3^ of normal tissue, whereas cryoablation treatment ablates 30 cm^3^ of normal tissue (68). The location of the tumor within the kidney plays a critical role in treatment success, as centrally located tumors more frequently fail effective tumor cell destruction (69–71). Treatment failure is also significantly associated with tumor size >4 cm (72).

#### Stereotactic Body Radiotherapy

Stereotactic body radiotherapy (SBRT) is a method of external beam radiotherapy that precisely targets tumors with high individual doses. Multiple 3D conformal beams or intensity-modulated RT ensure the delivery of highly conformal dose distribution with a steep gradient falloff at the tumor margin to minimize injury of surrounding normal tissue (73, 74). SBRT provides superior renal tumor control compared with conventional radiation therapy, both *in vitro* and *in vivo* (75–77). Despite concerns that radiation may accelerate mutational events, stereotactic treatment of VHL tumors (primary and metastatic RCC, as well as hemangioblastomas) showed no increase in tumor formation of surrounding tissue after doses of 30–40 Gy (78–81). Two reviews reported a local control rate of over 90% for large primary RCCs and up to 98% for small tumors (82, 83). The treatment is non-invasive, with a low toxicity and mild deterioration of renal function (84). Lesions close to collecting vessels are also amenable to therapy (85). Mild side effects included nausea, fatigue, skin rash, and local pain. Similar to other ablative therapies, assessment of the long-term efficacy is limited by the short follow-up interval and small study size.

In summary, because of the lack of comparing long-term studies, partial nephrectomy for tumors of 3–4 cm diameter is still the standard of care. However, minimal-invasive physically treatment with RFA, MWA, cryoablation, and SBRT carries certain treatment advantages. The choice of resection versus ablative treatment is dependent upon the tumor localization, treatment availability, and experience of the VHL center.

### Treatment of Advanced ccRCC

Advances in imaging and localized therapy have greatly improved detection and survival rates for VHL patients with early ccRCC. Currently, metastatic ccRCC is difficult to cure despite the availability of multiple systemic therapies. Before 2005, systemic therapy consisted of cytokine therapy with interleukin-2 and interferon-alpha, which was marked by severe toxicity and low response rates. Drugs that target the VHL–HIF–vascular endothelial growth factor (VEGF) pathway such as VEGF receptor inhibitors (sunitinib, sorafenib, pazopanib, and axitinib) significantly improved outcome (86–89). Bevacizumab, an anti-VEGF monoclonal antibody, was the first recombinant human monoclonal antibody that showed clinical efficacy in advanced disease (90). Today, sunitinib and pazopanib are approved first-line tyrosine kinase inhibitors for metastatic ccRCC.

Mechanistic target of rapamycin inhibitors like everolimus and temsirolimus aim at the “mechanistic target of rapamycin” complex mTORC1 which controls fundamental cellular functions such as growth, proliferation, and apoptosis. Intravenous temsirolimus was approved in 2007 following a study showing improved progressive free survival (PFS) compared with interferon alone or combination therapy with interferon and temsirolimus (91). For oral everolimus, PFS was longer in the treated group versus placebo group (92).

In 2016, cabozantinib was approved as a second-line inhibitor of VEGF receptor and a broad range of type III receptor tyrosine kinases. Both PFS and median overal survival were longer for cabozantinib treated patients versus everolimus treatment (93, 94). Levantinib, an oral multityrosine kinase, showed a synergistic effect with everolimus in patients previously pretreated for advanced ccRCC (95, 96).

Checkpoint inhibitors target “programmed cell death 1” (PD1), PD1-ligand, and cytotoxic T lymphocyte-associated protein 4 (CTLA-4) to activate T cell function. Nivolumab is a fully humanized IgG4 antibody against PD1, a negative regulator of T cell function, which improved survival in a subset of patients (97). Regardless of the targeted pathway, single agent treatment shows limited efficacy, with eventual treatment failure. Building on clinical experience favoring multiagent therapy (98), ongoing clinical trials are investigating combinations of kinase inhibitor treatments with immunotherapy, as well as combinations of immune modulators nivolumab and ipilimumab, an inhibitor of CTLA-4. A regression of metastases was observed after stereotactic radiotherapy in 4 of 28 renal cell carcinoma patients (99), suggesting that radiotherapy could enhance an immune response (98). Clinical trials testing the combination of radiotherapy with immunotherapy or targeted therapy are in progress (NCT02781506, NCT02019576, and NCT02978404).

Therapeutic options of advanced ccRCC have rapidly advanced over the past decade. Effective agents in advanced ccRCC have now been tested in earlier ccRCC stages and become appropriate first-line therapy drugs (100). Ideally, these drugs could serve as candidate perioperative agents with the potential to optimize postoperative outcome (101). In terms of precision medicine, the challenge now is to match a given patient with the optimal therapeutic agents with the help of robust molecular biomarkers.

## Therapeutic Targets and Future Trials

Traditional chemotherapy and conventional radiotherapy, unspecifically directed against highly proliferative cells, is poorly effective in ccRCC. Multiple dysregulated signaling pathways have recently been identified and therapies that target these pathways have shown clinical efficacy in a subset of patients. ccRCCs are highly vascular and treated by currently approved anti-angiogenic agents. Downstream components of the VHL–HIF–VEGF pathway are modulated by receptor tyrosine kinase inhibitors (axitinib, cabozantinib, levantinib, pazopanib, sorafenib, and sunitinib), as well as anti-VEGF monoclonal antibodies (bevacizumab). The PI3K/Akt/mTOR pathway is targetable by mTOR inhibitors (everolimus, temsirolimus), which reduces the accumulation of HIF protein.

Genomic profiling is a potential guide for treatment and prognosis. No predictive biomarkers have been validated for selecting treatment, but several candidate biomarkers for treatment response have been identified through retrospective patient studies using tumor DNA. For example, mutations in *MTOR, TSC1*, or *TSC2* were associated with response to mTOR inhibitors, 21% in responders versus 11% in non-responders (102). But many responders (56%) had no mTOR pathway mutation. A positive response to first-line everolimus was associated with *PBRM1* mutations, and a negative response with *BAP1* mutations. In addition, *KDM5* mutations were associated with better response with first-line sunitinib than with everolimus (103). These genomic biomarkers are currently being evaluated in prospective studies. Other potential biomarkers include RNA sequencing to detect structural rearrangement and transcription levels, microRNA sequencing, DNA methylation profiling, and metabolomics.

Given the inherent intratumor heterogeneity of ccRCC, therapy that targets truncal events involving *VHL* or chromosome 3p may be more effective than targeting subclonal pathways. *VHL* and *TCEB1* are mutually exclusive mutations in ccRCC, resulting in VHL complex degradation and HIFα stabilization. HIF-2α, constitutively activated in ccRCC, is mainly expressed in renal, lung, hepatic, and endothelial cells. Although transcription factors are difficult to target, a novel HIF-2α inhibitor PT2399 was recently developed which prevents binding of HIF-2α to ARNT/HIF-1β to activate a HIF-responsive promoter. Treatment with a HIF-2α inhibitor was investigated by grafting human ccRCC cell lines or patient tumor cells into nude mice, and by inactivating *vhl* in a zebrafish model.

Specific HIF-2α inhibition resulted in tumor regression in a subset of ccRCC cell line xenografts in mouse models of primary and metastatic ccRCC, in 10 of 18 patient-derived RCC xenografts in mice, and a patient with extensively pretreated metastatic ccRCC, who remained progression free for 11 months. Sensitivity to PT2399 correlated with a higher level of HIF-2α expression, and the presence of p53 (104, 105). Clinical trials with the HIF-2α inhibitor PT2385 are currently in progress (NCT02293980 and NCT03108066). Given the diversity of response, predictive biomarkers, such as HIF-2α and p53, may be useful for effective, targeted treatment. In zebrafish, treatment of *vhl^−/−^* embryos with a specific HIF2α inhibitor rescued pronephric abnormalities similar to human precancerous disease (38).

## Conclusion

Genomic sequencing has revolutionized the understanding of ccRCC by identifying multiple driver genes beyond *VHL*. Genetically engineered animal models to investigate combinations of *VHL*, epigenetic, and other genes provide a powerful preclinical model for elucidating the biology of ccRCC, developing novel combinatorial therapies, and identifying candidate biomarkers for clinical validation. In particular, uncovering the molecular basis driving tumor heterogeneity and the role of epigenetic genes will identify new pathways for intervention. Insights from these model systems will be clinically applicable to both hereditary and sporadic ccRCCs.

Surveillance and surgery remain standard of care in early ccRCC, while ablative therapies provide options for alternative treatment. For early ccRCC, current recommendations for intervention based solely on size could be better informed by prognostic tumor markers indicating a potential for aggressive growth, progression, and metastasis. Non-invasive biomarkers in blood or urine would be ideal for surveillance to avoid tissue biopsy. For advanced disease, a multiagent approach is supported by both clinical and preclinical observations, and ongoing clinical trials are currently in progress to evaluate treatment regimens and prognostic genomic biomarkers. In the future, genomic profiling is likely to be augmented by transcriptional and metabolomics analysis, as well as DNA methylation status. The efficacy of targeted therapy informed by tumor profiling may be limited by intratumor and intertumor heterogeneity. Immunotherapy has the potential to circumvent the high mutational load; clinical trials are in progress with single agent and multimodal therapy, targeting multiple signaling pathways or enhancing the immune response with stereotactic radiation.

## Author Contributions

Both authors made substantial contribution to the work and approved the final version for publication.

## Conflict of Interest Statement

The authors declare the absence of any commercial or financial relationships that could be construed as a potential conflict of interest.
